# Functional Interactions Between Recombinant Serum Amyloid A1 (SAA1) and Chemokines in Leukocyte Recruitment

**DOI:** 10.3390/ijms26052258

**Published:** 2025-03-03

**Authors:** Jo Van Damme, Sofie Struyf, Paul Proost, Ghislain Opdenakker, Mieke Gouwy

**Affiliations:** Laboratory of Molecular Immunology, Rega Institute for Medical Research, KU Leuven, 3000 Leuven, Belgium; jo.vandamme@kuleuven.be (J.V.D.); sofie.struyf@kuleuven.be (S.S.); paul.proost@kuleuven.be (P.P.); ghislain.opdenakker@kuleuven.be (G.O.)

**Keywords:** plasma chemokines, SAA1 fragments, induction, proteases, identification, leukocyte recruitment, synergy, inflammation, receptors, contamination

## Abstract

The acute phase response is a hallmark of all inflammatory reactions and acute phase reactants, such as C-reactive protein (CRP) and serum amyloid A (SAA) proteins, are among the most useful plasma and serum markers of inflammation in clinical medicine. Although it is well established that inflammatory cytokines, mainly interleukin-1 (IL-1), interleukin-6 (IL-6), and tumor necrosis factor-α (TNF-α) induce SAA in the liver, the biological functions of elicited SAA remain an enigma. By the classical multi-step protein purification studies of chemotactic factors present in plasma or serum, we discovered novel chemokines and SAA1 fragments, which are induced during inflammatory reactions. In contrast to earlier literature, pure SAA1 fails to induce chemokines, an ascribed function that most probably originates from contaminating lipopolysaccharide (LPS). However, intact SAA1 and fragments thereof synergize with CXC and CC chemokines to enhance chemotaxis. Natural SAA1 fragments are generated by inflammatory proteinases such as matrix metalloproteinase-9 (MMP-9). They mediate synergy with chemokines by the interaction with cognate G protein-coupled receptors (GPCRs), formyl peptide receptor 2 (FPR2) and (CC and CXC) chemokine receptors. In conclusion, SAA1 enforces the action of many chemokines and assists in local leukocyte recruitment, in particular, when the concentrations of specifically-induced chemokines are still low.

## 1. Introduction

Blood plasma is a rich source of inflammatory proteins secreted by various cells. These include cells specialized for the production of these proteins, such as liver hepatocytes, as well as common tissue cell types, such as fibroblasts, blood vessel smooth muscle and endothelial cells, circulating blood leukocytes and platelets. In many cases, the blood concentrations of these immunological mediators increase drastically after bacterial or viral infection as part of the common subsequent acute phase response and or in particular circumstances, such as the cytokine storms [1,2,3,4]. Cytokines and chemokines (chemotactic cytokines) are induced in various cell types by viral or bacterial toll-like receptor (TLR) ligands, such as double-stranded RNA, peptidoglycans or lipopolysaccharide (LPS) [5,6,7]. Cytokines may further induce chemokines and other cytokines in various tissues, as well as acute phase proteins, including SAA, in the liver [2,4,8,9,10]. As a consequence, cytokines interact in a complex network of molecules regulating the immunological responses. For example, bacterial LPS, through interactions with TLR4, induces the production of interleukin-1 (IL-1) in blood monocytes [11], which, in turn, induces IL-6 and chemokine production in fibroblasts and endothelial cells [12,13,14,15,16,17,18]. Furthermore, both IL-1 and IL-6 are potent stimulators of the acute phase response leading to SAA secretion by the liver [19,20,21,22]. Here, we focus on the interaction between chemokines and SAA in the attraction of leukocytes to sites of infection.

Most plasma proteins, including immunoglobulins, have been identified by the traditional protein purification technology based on size, charge, and affinity for specific ligands as their different biochemical properties. For cytokines with high specific biological activity, and hence, detectable in plasma only in the picogram range, the isolation was more difficult. Their identification was often based on the in vitro-induced production on a large scale by stimulated cells and subsequent chromatographical separation with the use of activity assays for their detection. For example, interferons were the first identified cytokines based on their antiviral activity [23,24]. A major burden in the process of cytokine identification was the need to purify these molecules from a pool (defined ‘soup’) of proteins to chromatographical homogeneity and to retain sufficient amounts of protein after multiple purification steps to allow for the identification of their primary structure by amino acid sequencing [25,26,27,28,29,30,31]. Furthermore, contaminating molecules, such as bacterial LPS or lipoproteins interfering at picogram levels in the biological test assays, which are used to isolate and biologically characterize purified cytokines, can lead to artefactual observations, and hence false conclusions (vide infra) [32,33,34]. The possible impact of LPS contamination on the structure–function relationship of natural hepatocyte SAA will not be discussed here [35].

## 2. Isolation and Identification of Chemotactic Factors from Blood Plasma

Chemokines constitute a large family of low molecular mass chemotactic proteins characterized by the conservation of four cysteine residues in their primary structure. Depending on the positioning of the cysteines in the NH_2_-terminal region, these proteins are classified as CXC or CC chemokines [36,37]. Chemokines exert their biological activity through binding and signaling via G protein-coupled CXC and CC chemokine receptors (CXCR and CCR) expressed on their target cells. A particular chemokine can recognize several receptors and a single receptor can bind to different chemokines rendering a complex network of ligand–receptor interactions. Furthermore, different leukocyte types (e.g., granulocytes, monocytes, lymphocytes) express a distinct set of chemokine receptors [38,39,40,41,42,43,44,45,46].

Initially, a number of chemokines have been identified through their isolation from natural cellular sources, i.e., conditioned media from in vitro stimulated cells meticulously cultured at a large scale, followed by tedious and thorough purification and amino acid sequencing of the proteins exerting chemotactic activity [26,30,38,47,48,49,50,51]. Additional chemokine structures were identified through molecular cloning technologies or discovered via homology searches after the sequencing of the human genome was accomplished [51,52,53]. In contrast to the chemokines isolated from natural cellular sources and characterized based on a specific biological activity combined with protein purification, recombinant chemokines expressed in prokaryotic cells are required to be biologically tested for chemotactic activity in order to identify possible target cells and to be classified as active chemokines. The contamination of bacterially expressed chemokine with exogenous interfering substances such as LPS is a risk factor for false conclusions during their biological characterization [32,33,34,54,55,56]. In addition, human cells cultured for the production of natural chemokine are typically grown in the presence of bovine serum, which implies the risk of copurification of animal chemotactic factors from the serum-containing conditioned cellular medium [57,58,59]. Therefore, bovine serum was also directly analyzed for its content of chemotactic factors [29,30,60].

In our laboratory, we used bovine serum, commercially available for in vitro cell culture to demonstrate the presence—in low concentrations—of cytokines and chemokines. Such animal serum, present in the conditioned medium for cell cultures, was concentrated and enriched for relevant proteins by adsorption to controlled pore glass beads or silica matrix as initially demonstrated for the purification of interferons and interleukins (see above). As a second purification step, chemotactic factors for different leukocyte types were selectively extracted based on their affinity for heparin. This chromatographical approach with salt gradient elution and fractionation already allowed for the separation of distinct chemotactic entities corresponding to different peaks containing biological activity in the elution profile. Further purification of distinct so-called peaks of activity by cation exchange chromatography and/or reversed-phase HPLC yielded several pure chemotactic proteins as illustrated in Table 1. These proteins were tested for homogeneity by SDS-PAGE under reducing conditions and were visualized by silver staining before the identification of their NH_2_-terminal sequence by Edman degradation [29,30,60].

Similar to the human system, substantial amounts of thrombocyte-derived platelet factor-4 (PF-4/CXCL4) were recovered from bovine serum, which is probably released during blood coagulation (unpublished data). CXCL4 has poor chemotactic activity for leukocytes but exerts angiostatic activity on endothelial cells [61,62]. In contrast, the presence of macrophage inflammatory protein-1α (MIP-1α/CCL3) in bovine serum was rather unexpected since this chemokine is produced by leukocytes during an inflammatory response to infection. CCL3 is the most potent chemoattractant for monocytes and immature dendritic cells (minimal effective concentration of 0.03 ng/mL) via signaling through its CCR1 and CCR5 receptors [63,64,65]. Completely unexpected was the discovery of a novel CC chemokine designated Regakine-1, for which no human homolog has yet been identified. Although abundantly present in bovine serum, Regakine-1 has only weak chemotactic activity for lymphocytes and granulocytes [29,51]. However, Regakine-1 allowed for the discovery of the chemokine synergy phenomenon, since it enhances the chemotactic response towards low suboptimal concentrations of various CXC and CC chemokines [66]. Finally, the isolation of a COOH-terminal fragment of bovine SAA1 based on chemotactic activity was a surprise, although such effect had previously been ascribed to intact human SAA1 [60,67]. Indeed, intact human SAA1 chemoattracts granulocytes and monocytes via the formyl peptide receptor 2 (FPR2) [34,68,69]. Similar to Regakine-1, SAA1(46-112) was found to synergize with CCL3 and CXCL8 in monocyte and granulocyte chemotaxis, respectively [60,66,70,71]. The G protein-coupled receptor FPR2 is also implicated in this synergistic effect with CCR and CXCR ligands [72,73].

## 3. Commercial Recombinant Serum Amyloid A1 (SAA1) Purified to Homogeneity Fails to Induce Inflammatory Mediators

Numerous studies are dealing with the capacity of SAA1 to stimulate the expression of inflammatory mediators in mononuclear leukocytes, fibroblasts, and endothelial cells [68,74,75,76,77]. Indeed, commercially available recombinant SAA1 has been reported to induce cytokines such as IL-1, IL-6, IL-10, and TNF-α, as well as a number of chemokines including CXCL1, CXCL8, CCL2, and CCL3 [68,76,77,78,79,80,81,82,83]. This would indicate that hepatocyte stimulating factors such as IL-1 and IL-6 induce SAA1 in the liver and that upon appearance in the blood circulation, SAA1 can, in return, induce these cytokines in monocytes and endothelial cells. Although such positive feedback loop would make sense to amplify the acute phase response, one may wonder which mechanism is controlling this process. Moreover, SAA1 is reported to stimulate the production of pro-inflammatory CXC and CC chemokines in parallel with its inducers IL-1 and TNF-α, which would imply another amplification pathway of the inflammatory response [4,77]. A number of putative receptors implicated in the biological activities of SAA1 have been reported: RAGE, SR-BI, TLR2, TLR4, and FPR2 [67,78,82,84,85,86,87,88,89,90,91,92,93]. However, cytokines induce chemokines via their proper specific cellular receptors, whereas SAA1 predominantly exerts these activities via TLR2 and TLR4 [78,82,86,89,90,91]. Since TLRs are well known to respond to very low levels of microbial products with the induction of cytokines and chemokines, one has to be careful in discriminating true SAA1 activity from activities of possible contaminants. In particular, sufficient attention needs to be paid with the use of recombinantly produced SAA1 in bacteria [33,34]. Indeed, commercial recombinant *E. coli*-derived SAA1 has been used in most studies that report the cytokine/chemokine induction properties of the SAA mentioned above. Such bacteria-derived preparations may still contain considerable amounts of LPS, which is the most potent cytokine and chemokine inducer. It was suggested that the above-mentioned proinflammatory effects ascribed to SAA1 are in fact mediated by contaminating bacterial products such as LPS and lipoproteins [33,34]. To exclude this artefact, we purified commercially available recombinant SAA1 to homogeneity by reversed-phase chromatography procedures and confirmed the protein identity by mass spectrometry [34]. In such homogeneous SAA1 preparations, the LPS content remained under the detection limit as tested in the Limulus amoebocyte lysate assay [34]. As shown in Table 2, commercially available recombinant SAA1 was able to induce chemokines in LPS-sensitive monocytes already at 10 ng/mL, whereas homogeneously purified (recombinant) SAA1 failed to achieve this at 1000 ng/mL. This indicates that this effect, which is only observed with insufficiently purified commercial SAA1, is in all probability mediated by TLR2- and TLR4-responsive contaminants. In fact, Burgess et al. could block the production of TNF-α from macrophages by lipoprotein lipase [33].

In addition, other reported effects of SAA1 mediated by TLR signaling were investigated. Indeed, it has been shown that SAA1 can upregulate the expression of matrix metalloproteinases (MMPs) such as MMP-1 and MMP-9 [81,94,95,96]. Table 2 demonstrates that, upon purification to homogeneity, SAA1 lost its MMP-inducing capacity in monocytes [34,97]. Similarly, the capacity of pure SAA1 to induce the production of reactive oxygen species in monocytes is also more than 10-fold reduced compared to impure SAA1 [34]. It can be concluded that more scrutiny is needed in the interpretation of published data on SAA1 activities mediated via TLRs.

## 4. The Revised Cytokine-Chemokine-Serum Amyloid A1 (SAA1) Network

**Figure 1:**
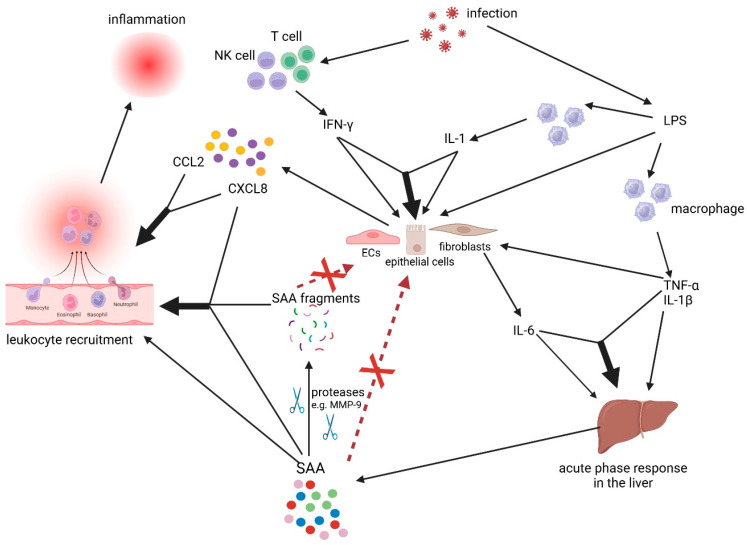
Synergistic interactions between cytokines, chemokines, and SAA in the inflammatory response. Cytokines interact in a complex network of molecules regulating immunological responses. During an infection, bacterial lipopolysaccharide (LPS) stimulates macrophages to produce TNF-α and IL-1β, which, in turn, induce IL-6 and chemokine production in fibroblasts and endothelial cells (ECs). Interferons, e.g., IFN-γ produced by T cells and natural killer (NK) cells during an infection, synergize with IL-1 (bold black arrow) to induce chemokines in epithelial cells and fibroblasts. Furthermore, IL-6 on its own or in synergy (bold black arrow) with TNF-α and/or IL-1β is a potent stimulator of the acute phase response leading to liver acute phase proteins (e.g., SAA). Upon secretion, SAA1 undergoes posttranslational processing by various proteinases including MMPs (e.g., MMP-9). In contrast to commercial recombinant intact SAA1, none of these SAA1 peptides nor the pure intact SAA1 were able to induce CXC and CC chemokines in various cell types [e.g., monocytes, endothelial cells, fibroblasts] (red dotted line). The induced chemokines subsequently synergize to stimulate the migration of leukocytes (e.g., monocytes, neutrophils, DC) to the site of inflammation (bold black arrow). Furthermore, both intact pure SAA1 and SAA1 fragments can synergize (bold black arrows) with chemokines to enhance leukocyte migration to inflammatory foci. Standard arrows indicate production by or action on indicated cells (created by BioRender).

Based on recent findings that the so-called cytokine- and chemokine-inducing capacities of SAA1 are merely due to contaminating microbial LPS and lipoproteins, the interactive network of these inflammatory mediators becomes less complex. Nevertheless, the known cascade of events to develop an acute phase response remains solid: bacterial LPS induces in TLR-expressing monocytes the alarm cytokines (endogenous pyrogens) IL-1 and TNF-α, which are potent inducers of secondary cytokines (sometimes resulting in a cytokine storm) such as IL-6, as well as chemokines in tissue fibroblasts, blood vessel endothelial cells, and epithelial cells (Table 3 and Figure 1) [11,13,15,16,17,18]. All these inflammatory mediators exert their biological activities through specific though distinct receptors expressed on specific target cells. By binding to their different GPCRs, chemotactic cytokines selectively attract various leukocyte types including granulocytes (CXCR1 and CXCR2 activation by CXCL8), monocytes (by CCL2 on CCR2) and lymphocytes (by CXCL9 and CXCL10 on CXCR3 and CCL3 on CCR1 and CCR5) to the inflammatory site. Furthermore, IL-1 and IL-6 collaborate to stimulate liver hepatocytes to secrete acute phase proteins including SAA1, which has been found to also attract leukocytes albeit via binding to another type of GPCR, i.e., FPR2 [19,20,21,22,34,67]. However, the above-mentioned positive endogenous amplification loop by SAA1 as a cytokine and chemokine inducer acting via TLRs seems unlikely to occur in vivo, since it is based on artefactual LPS contamination. It is thereby not excluded that the acute phase response is persisting in vivo as long as LPS is present and TLR-responsive monocytes are available.

## 5. Synergistic Interaction Between Recombinant Serum Amyloid A1 (SAA1) and Chemokines in Leukocyte Migration

A positive interaction between cytokines leading to enhanced biological activity has been reported on numerous occasions (Figure 1). For example, IL-1 and interferons synergize to induce the chemokines CCL2 and CCL8 in fibroblasts and epithelial cells [15,98]. Similarly, IL-1 and IL-6 cooperate for the induction of SAA in hepatocytes [22,99,100].

More recently, a synergistic interaction between chemokines in leukocyte migration was first described for Regakine-1 (Table 1) [29,66]. Indeed, since the CC chemokine Regakine-1 is constitutively present in the blood circulation but is lacking potent chemotactic activity, it was speculated that it could function as a natural chemokine antagonist to dampen the inflammatory response by competing for binding to the receptors of potent chemoattractants. However, the opposite observation was made, in that Regakine-1 cooperated with CXCL8 and CCL7 to enhance the chemotactic response of granulocytes and lymphocytes, respectively [29,66]. This principle turned out to be applicable to a number of inflammatory members within the chemokine family. This phenomenon is probably mediated by receptor binding since most if not all chemokines bind to distinct GPCRs for signal transduction. In general, a weak chemotactic response to a suboptimal concentration of a granulocyte CXC chemoattractant (CXCL8) is enhanced by a supra-optimal concentration of a mononuclear cell CC chemoattractant (CCL2) and vice versa [101].

In addition to Regakine-1, the bovine SAA1 (46-112) fragment was isolated from blood plasma based on weak chemotactic activity for granulocytes and monocytes. However, the chemotactic effects of this SAA fragment and its human equivalent SAA1 (47-104) are significantly enhanced in the presence of suboptimal concentrations of CXCL8 and CCL3 to attract granulocytes and monocytes, respectively. Intact SAA1 also synergized with these chemokines and the effect could be desensitized by the pretreatment of the cells with the SAA1 (47-104) fragment, indicative of a GPCR-mediated phenomenon. The implication of FPR2, the known GPCR used by SAA1 for chemotaxis, was evidenced by the blockade of the synergy between SAA1 and CXCL8 or CCL3 in chemotaxis by WRW4, a specific FPR2 antagonist [60].

SAA1 is a poor agonist for FPR2 requiring a suboptimal dose of a potent GPCR agonist (CXCL8) to obtain a solid chemotactic response. SAA is thus considered to be a chemokine synergist rather than a true chemotaxis agonist. But why is the impure SAA1 more potent since LPS has no direct chemotactic effect? It was observed that LPS can also synergize with authentic chemokines in the in vitro chemotaxis assay, suggestive of a TLR/GPCR alliance [102]. Indeed, LPS can rapidly induce the production of low levels of active chemokine in the leukocytes subjected to the chemotaxis test, allowing for synergy, thereby mimicking a potent direct chemotactic effect of SAA1 [102].

## 6. Serum Amyloid A1 (SAA1) Fragments: Generation by Matrix Metalloproteinases (MMPs), Chemotactic Properties, and Mode of Action

Serum amyloid A is an acute phase protein induced to extremely high concentrations during chronic [103,104] and acute [105] inflammation, such as in patients with inflammatory bowel disease [106]. Similar to many chemokines, SAA1 undergoes posttranslational processing by various proteinases upon secretion including MMPs and cathepsins [107,108,109,110]. In particular, SAA1 is cleaved by MMP-1 into the NH_2_-terminal SAA1 (1-57) and the COOH-terminal SAA1 (58-104) fragments, whereas MMP-2 generates SAA1 (1-51) and SAA1 (52-104). SAA1 is processed by MMP-3 into both SAA1 (57-104) and SAA1 (58-104). MMP-9 generates the SAA1 (57-104) fragment, but no NH_2_-terminal counterpart could be recovered, suggestive of the potential degradation of that part of the molecule [69,108]. The cysteine protease cathepsin B cleaves SAA into multiple fragments at different sites within the molecule [109,111].

In the search for novel chemotactic factors present in blood plasma, it was a surprise to isolate a 7.3 kDa COOH-terminal fragment of SAA based on the purification strategy and biological test systems for chemokines [60]. In order to investigate their biological properties, this COOH-terminal SAA1 (47-104) fragment and the MMP-9-derived SAA1 (52-104) and SAA1 (58-104) were chemically synthesized and purified to homogeneity. In contrast to the commercial recombinant intact SAA1, none of these COOH-terminal SAA1 peptides, nor the NH_2_-terminal part SAA1 (1-51) were able to induce chemokine production in monocytes or fibroblasts [69]. As indicated above, this latter effect of the recombinant intact SAA1 is most probably TLR-mediated by the LPS contamination of the recombinant product. Indeed, further purified recombinant intact SAA1, similar to the chemically synthesized SAA1 peptides, also failed to act as a chemokine inducer in fibroblasts or monocytes [34,60,69]. Furthermore, pure intact SAA1 and synthetic SAA1 fragments lack significant direct chemotactic activity for granulocytes in vitro [34,69]. However, both intact and COOH-terminal SAA1 fragments potentiate the granulocyte chemotactic effect of CXCL8, whereas the NH_2_-terminal counterpart SAA1 (1-51) failed. At µg concentration, SAA1 (58-104) synergized with CXCL8 at suboptimal chemokine concentration (ng range) to chemoattract granulocytes, whereas SAA1 (1-51) did not [69]. During the inflammatory response, cytokines such as IL-1 and IL-6 upregulate the production of SAA1 drastically (up to 1000-fold), potentially reaching mg/mL concentrations in the blood circulation. Simultaneously, proteases such as MMP-9 are released from granules in granulocytes as well as de novo induced by IL-1 in monocytes, yielding a sufficient and stable amount of MMP-9 that can cleave SAA efficiently in fragments including SAA (58-104) [69], which should be generated in mg/mL concentrations in blood and inflammatory exudates. Thus, the concentrations of SAA fragments at µg/mL (cfr Table 2) can be reached in vivo and are relevant for synergy with chemokines in leukocyte chemotaxis.

Other biological activities of SAA1 are reportedly mediated by binding to and signaling through unrelated receptors, including RAGE, SR-BI, and SR-BII [84,85,87,88,92,93]. Similar to chemokines, SAA1 exerts its chemotactic activity for leukocytes by binding to a GPCR, i.e., FPR2 [67]. The synergistic chemotactic effect in granulocyte migration between intact SAA1 or its COOH-terminal peptides with CXCL8 could be blocked by the selective FPR2 antagonist WRW4 [34,69].

## 7. Conclusions: So-Called Recombinant Serum Amyloid A1 (SAA1) Activities Under the Pressure of Contaminants

It has long been considered technically impossible to purify minute amounts of cytokines/chemokines to homogeneity from a natural cellular source (conditioned medium) or body fluid (blood plasma) containing hundreds of different proteins, most of these (e.g., albumin, SAA) in much larger quantities than cytokines/chemokines. The biological characterization of such purified new inflammatory mediators was hampered by the presence of either already known or yet unidentified cytokines/chemokines exerting interfering activity even at the picogram range. Being aware of this danger and inherent risk of generating biologically false results, academic protein biochemists have constantly worked under the pressure of this “sword of Damocles”. In contrast, with the introduction of the molecular cloning technology, recombinant protein preparations became available upon expression in prokaryotic bacteria (e.g., *E. coli*) that were free of interfering biologically active proteins from eukaryotic origin. However, the subsequent technological boost in the commercial production of unlimited amounts of such new inflammatory mediators weakened the attention for the need to purify also recombinant proteins to homogeneity. In particular, bacterial products such as LPS, a molecule which is not detected as contaminant on SDS-PAGE gels and a number of other techniques, and lipoproteins are known to interfere at low concentrations with eukaryotic mediators in inflammatory responses. As described here, the contamination of recombinant commercial preparations of SAA with LPS and/or lipoproteins has led to biological activities incorrectly ascribed to recombinant SAA [34].

Our studies on chemotactic factors present in serum or plasma yielded several surprising novel insights about the regulation of inflammation in infections and the biology of the acute phase reactant SAA1. Earlier literature about the direct chemotactic activity of SAA1 mediated by TLR2 and TLR4 seems undermined by the fact that pure SAA1 protein preparations lack this activity and receptor activation, yet synergize with other chemokines through GPCRs. In all kinds of infections with either viruses, bacteria, fungi and parasites, the cytokines IL-1, IL-6, and TNF-α induce considerable amounts of SAA1 in the liver and minute amounts of specific chemokines at entry and propagation sites of infections. Intact SAA1 and fragments of SAA1, generated by, e.g., neutrophil proteinases (MMP-9 and cathepsins), synergize locally with subminimal or low concentrations of classical chemokines to recruit various leukocyte types in the fight against infections.

## Figures and Tables

**Table 1:** Biochemical and biological characteristics of chemotactic proteins purified and identified from bovine serum.

Properties	Chemotactic Factor			
	CXCL4/PF-4	CCL3/MIP-1α	Regakine-1	SAA1(46-112)
**Affinity for heparin**	high	moderate	moderate	low
**Molecular size (kDa)**	7	7.7	7.5	7.3
**Receptor usage**	ND	CCR1, CCR5	unknown	FPR2
**Target cells**	EC	monocytes, iDC	T cells, granulocytes	monocytes, granulocytes
**Serum concentration (ng/mL)**	high	10	100	ND
**Angiostatic/Chemotactic** **activity**	potent	very potent	synergy	synergy
**Synergizing chemokine**	ND	CXCL12	CCL7, CXCL6, CXCL7, CXCL8	CCL3, CXCL8

EC: endothelial cells; iDC: immature dendritic cells; ND: not determined.

**Table 2:** Biological properties of recombinant SAA1 purified to homogeneity by RP-HPLC.

Biotest System	Tested Cells	RP-HPLC-Purified rSAA1		Commercial rSAA1	
		MEC (ng/mL)	Result	MEC (ng/mL)	Result
**Chemokine (CXCL8) induction**	monocytes	>1000	neg	10	pos
**Chemokine (CCL3) induction**	monocytes	>300	neg	10	pos
**Proteinase (MMP-9) induction**	monocytes	>100	neg	10	pos
**ROS production**	monocytes	>1000	neg	100	pos
**Leukocyte recruitment after** **intra-articular injection**	mononuclear cells	100 ^a^	pos	100	pos
**Leukocyte recruitment after** **intra-articular injection**	granulocytes	100 ^a^	pos	100	pos
**Synergy (CXCL8) in chemotaxis**	granulocytes	3000	pos	300	pos
**Synergy (CXCL8) in shape change**	granulocytes	3000	pos	3000	pos
**Synergy (CXCL8) in actin polymerization**	granulocytes	300	pos	ND	ND

MEC: minimal effective concentration; ND: not determined; ROS: reactive oxygen species; neg: negative; pos: positive. ^a^ Intra-articular injection: 100 ng/injection site.

**Table 3:** Interactive production of cytokines, chemokines, and SAA in various cell types.

Producer Cell	Inducer	Produced Mediators				
		IL-1	IL-6	CXCL8	CCL2	SAA1
**Monocyte**	**LPS**	**+**	**+**	**+**	**+**	**+**
	**IL-1**		**+**	**+**	**+**	**-**
	**SAA1**	**-**	**-**	**-**	**-**	
**Fibroblast**	**LPS**	**-**	**-**	**-**	**-**	**-**
	**IL-1**		**+**	**+**	**+**	**-**
	**SAA1**	**-**	**-**	**-**	**-**	
**Hepatocyte**	**IL-1**		**+**	**+**	**+**	**+**
	**IL-6**	**-**		**-**	**-**	**+**
	**SAA1**	**-**	**-**	**-**	**-**	

**+**: induction; **-**: no induction. Table 3 summarizes published in vitro data from our laboratory [11,12,15,16,17,18,34,98].
